# Supplementary material for: Embedding security into ferroelectric FET array via in situ memory operation
Source: Nat Commun. 2023 Dec 13;14:8287. doi: 10.1038/s41467-023-43941-5 (PMC10719298; doi:10.1038/s41467-023-43941-5)
Supplement: Supplementary file 1 — Supplementary Information [file 41467_2023_43941_MOESM1_ESM.pdf]

Supplementary Materials

Measurement Setup

Fig. S1 shows our measurement setup for FeFET characterization, which utilize a PXI System that incorporates SMU and PPMU.

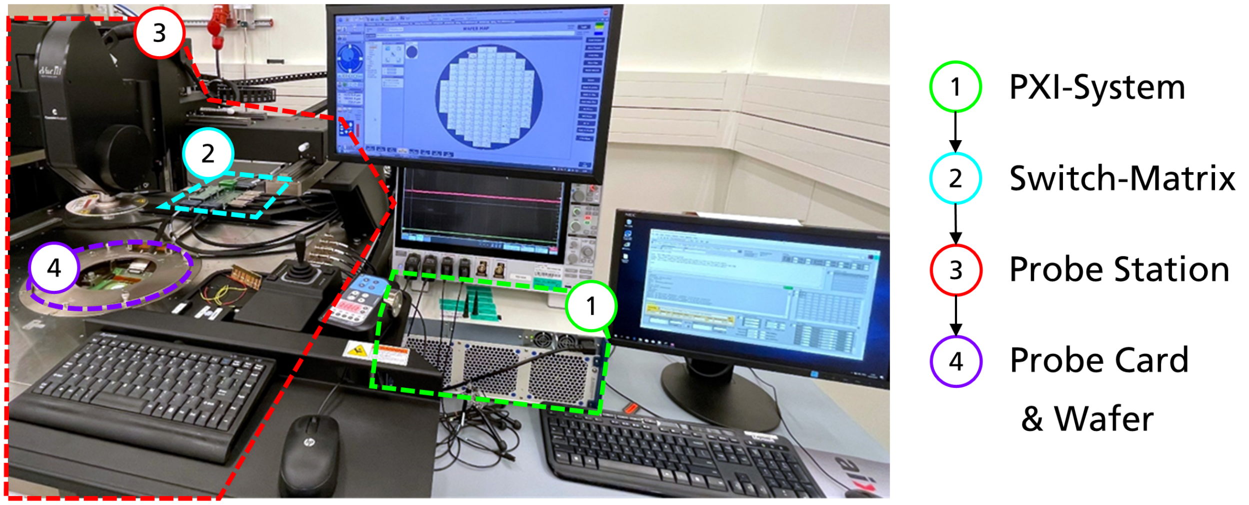

Figure S1: Measurement setup for FeFET characterization. The measurement setup utilizes a PXI System that incorporates Source Measurement Units (SMU) and Pin Parametric Measurement Units (PPMU). The PPMUs are employed to configure the Switch Matrix, allowing the source signals to be routed to the corresponding contact needles. The test structures, present on 300 mm wafers, are connected to the measurement setup through a semi-automatic probe station, facilitated by a probe card.

NAND encryption scheme

Besides the FeFET AND array, the proposed encryption scheme can be implemented in the form of FeFET NAND array which provides potentially higher integration density (shown in Fig. S2). Similar to the AND array case, 2 neighboring FeFETs are grouped as a cell to represent 1 bit stored information. For the encryption process, firstly the key is XORed with PT to obtain the CT. If CT is 1 (0), the two consecutive FeFETs on the selected NAND string are programmed to HVT and LVT (LVT and HVT) respectively. During the decryption process, two possible voltages (V_{r1} and V_{r2}) are applied on the gate nodes of FeFETs, which satisfy $V_{r1} > V_{\text{th,high}} > V_{r2} > V_{\text{th,low}}$. V_{r1}/V_{r2} are applied on the first/second FeFET when Key=0, and V_{r2}/V_{r1} are applied on the first/second FeFET when Key=1. If PT=1, V_{r1} is applied on the HVT FeFET and V_{r2} is applied on the LVT FeFET, in which case they are both ON so that a high current is sensed on the NAND string. If PT=0, V_{r1} is applied on the LVT FeFET and V_{r2} is applied on the HVT FeFET. Since the HVT FeFET is OFF, the read current is low. In this way, CT is XORed with Key so that PT is obtained by sensing the read current.

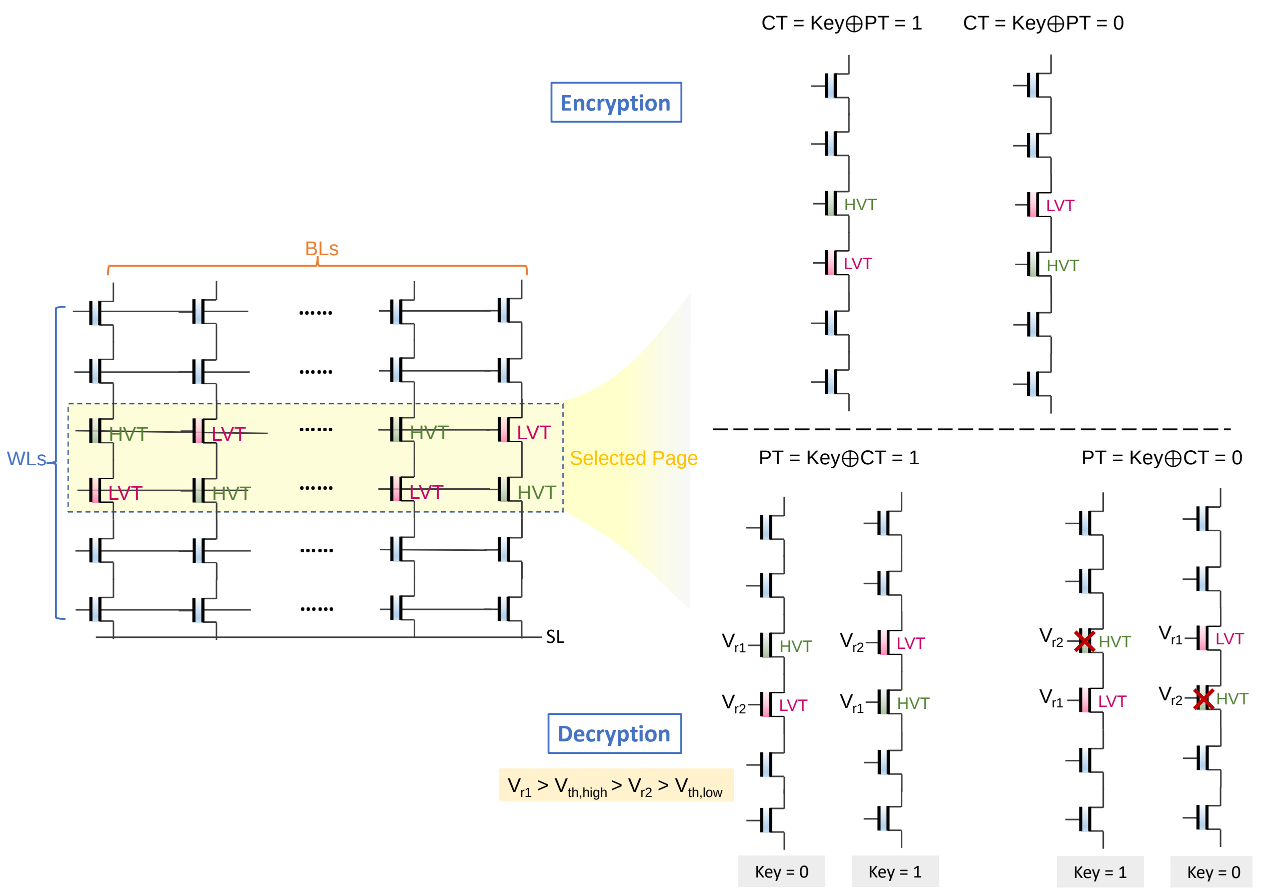

Figure S2: The encryption and decryption scheme for NAND memory arrays

NOR encryption scheme

The proposed encryption scheme can be implemented in the form of FeFET NOR array as well (shown in Fig. S3). Similar to the AND array case, 2 consecutive FeFETs in the same column are used to represent 1 bit encrypted information. During the encryption process, after the Key is XORed with PT to obtain the CT, the top and bottom FeFET are programmed to HVT (LVT) and LVT (HVT) respectively if CT=1 (0). While during the decryption process, the read voltage ($V_{\text{th,high}} > V_{\text{R}} > V_{\text{th,low}}$) is applied on the top (bottom) FeFET if Key=1 (0). Only when V_R is applied on the LVT FeFET, a high current is sensed which represents PT=1. In this way, the XOR operation between Key and CT is realized in the NOR array.

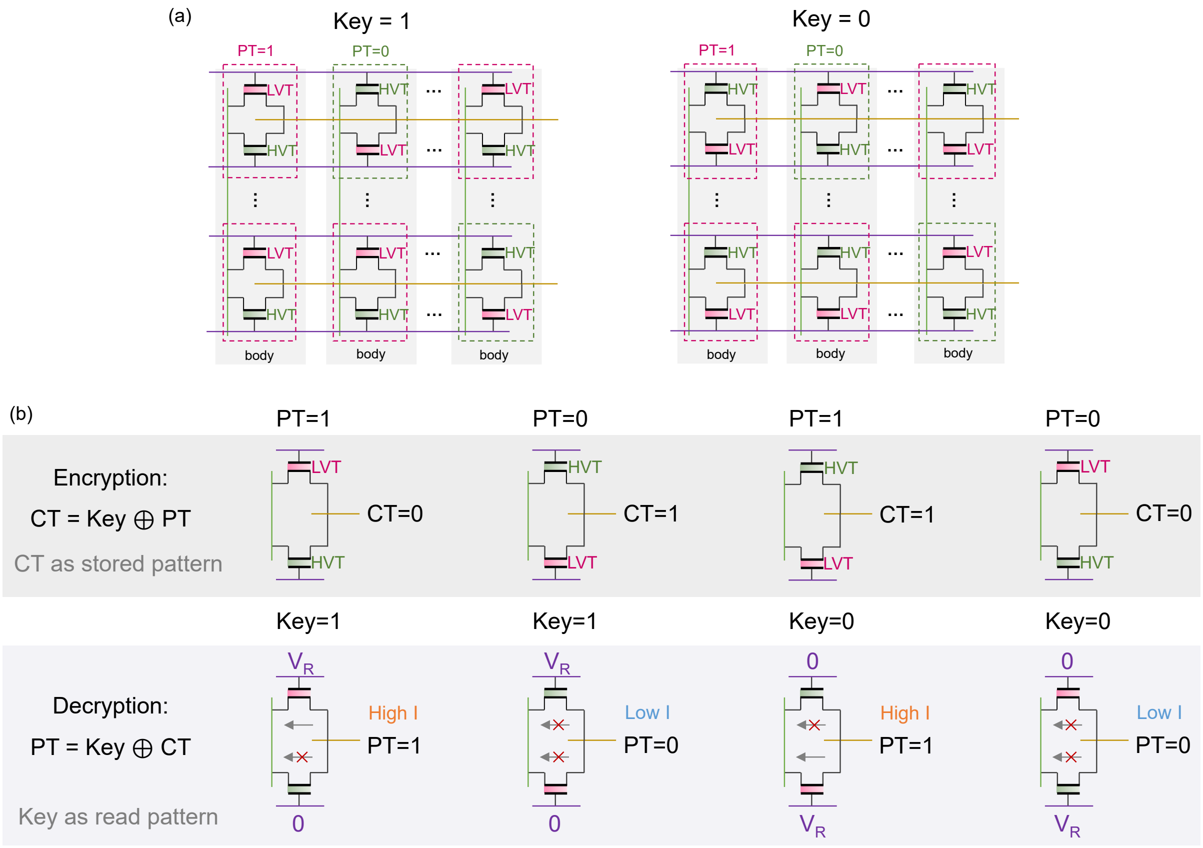

Figure S3: The encryption and decryption scheme for NOR memory arrays in (a) array level and (b) cell level.

Programming and inhibit scheme

Regarding the programming scheme, as shown in Fig.S4, we firstly program the whole block which need to be encrypted to HVT state by asserting all WLs at $-V_W$ (i.e., V_W=3.3 V in this work), then program the complementary FeFET in each 2FeFET cell to LVT state by applying $+V_W$ on each WLs. Therefore a total of 3 cycles are required to implement the encryption scheme. For proper operation of the array, inhibition bias schemes need to be applied to prevent undesired programming to unselected cells. Two schemes are generally available, i.e., $V_W/2$ and $V_W/3$ scheme[23]. Here, we choose $V_W/3$ scheme to minimize the disturb, as shown in Fig.S4.

Regarding the endurance of FeFETs used in arrays, though three programming cycles are required in encrypted FeFET cell, the programming is divided in the two FeFETs. Therefore, the number of writes is still the same as that in single FeFET array.

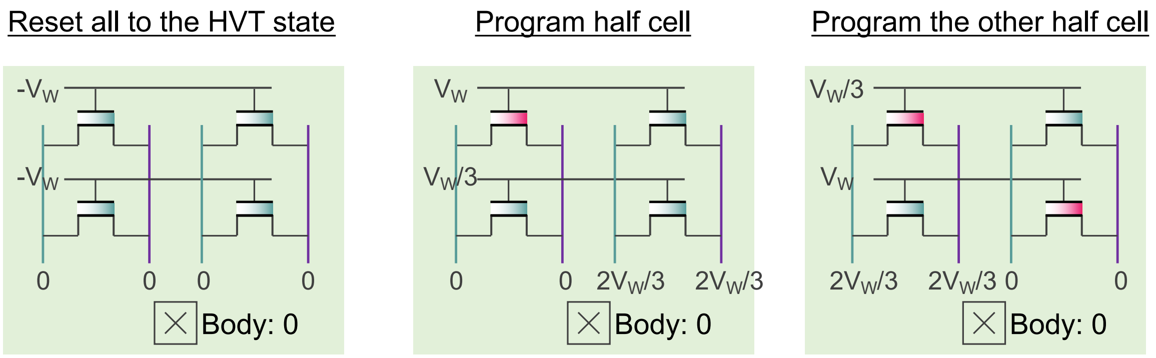

Figure S4: Three steps for programming of the FeFET AND array to implement the encryption scheme.
